# GMP-Compliant Manufacturing of NKG2D CAR Memory T Cells Using CliniMACS Prodigy

**DOI:** 10.3389/fimmu.2019.02361

**Published:** 2019-10-10

**Authors:** Lucía Fernández, Adrián Fernández, Isabel Mirones, Adela Escudero, Leila Cardoso, María Vela, Diego Lanzarot, Raquel de Paz, Alejandra Leivas, Miguel Gallardo, Antonio Marcos, Ana Belén Romero, Joaquín Martínez-López, Antonio Pérez-Martínez

**Affiliations:** ^1^Hematological Malignancies H12O, Clinical Research Unit, Spanish National Cancer Research Centre (CNIO), Madrid, Spain; ^2^Translational Research in Pediatric Oncology, Hematopoietic Transplantation and Cell Therapy, IdiPAZ, Hospital Universitario La Paz, Madrid, Spain; ^3^Pediatric Molecular Hemato-Oncology Department, Instituto de Genética Médica y Molecular (INGEMM), Hospital Universitario La Paz, Madrid, Spain; ^4^Applications Department, Miltenyi Biotec S.L., Madrid, Spain; ^5^Hematology Department, Hospital Universitario La Paz, Madrid, Spain; ^6^Hematology Department, Hospital Universitario12 de Octubre, Madrid, Spain; ^7^Pediatric Hemato-Oncology Department, Hospital Universitario La Paz, Madrid, Spain

**Keywords:** NKG2D CAR, memory T cells, automated production, large-scale, clinical-grade, CliniMACS prodigy

## Abstract

Natural killer group 2D (NKG2D) is a natural killer (NK) cell-activating receptor that recognizes different stress-induced ligands that are overexpressed in a variety of childhood and adult tumors. NKG2D chimeric antigen receptor (CAR) T cells have shown potent anticancer effects against different cancer types. A second-generation NKG2D CAR was generated by fusing full-length human NKG2D to 4-1BB costimulatory molecule and CD3ζ signaling domain. Patient-derived CAR T cells show limitations including inability to manufacture CAR T cells from the patients' own T cells, disease progression, and death prior to return of engineered cells. The use of allogeneic T cells for CAR therapy could be an attractive alternative, although undesirable graft vs. host reactions may occur. To avoid such adverse effects, we used CD45RA^−^ memory T cells, a T-cell subset with less alloreactivity, as effector cells to express NKG2D CAR. In this study, we developed a protocol to obtain large-scale NKG2D CAR memory T cells for clinical use by using CliniMACS Prodigy, an automated closed system compliant with Good Manufacturing Practice (GMP) guidelines. CD45RA^+^ fraction was depleted from healthy donors' non-mobilized apheresis using CliniMACS CD45RA Reagent and CliniMACS Plus device. A total of 10^8^ CD45RA^−^ cells were cultured in TexMACS media supplemented with 100 IU/mL IL-2 and activated at day 0 with T Cell TransAct. Then, we used NKG2D-CD8TM-4-1BB-CD3ζ lentiviral vector for cell transduction (MOI = 2). NKG2D CAR T cells expanded between 10 and 13 days. Final cell products were analyzed to comply with the specifications derived from the quality and complementary controls carried out in accordance with the instructions of the Spanish Regulatory Agency of Medicines and Medical Devices (AEMPS) for the manufacture of investigational advanced therapy medicinal products (ATMPs). We performed four validations. The manufacturing protocol here described achieved large numbers of viable NKG2D CAR memory T cells with elevated levels of NKG2D CAR expression and highly cytotoxic against Jurkat and 531MII tumor target cells. CAR T cell final products met release criteria, except for one showing *myc* overexpression and another with viral copy number higher than five. Manufacturing of clinical-grade NKG2D CAR memory T cells using CliniMACS Prodigy is feasible and reproducible, widening clinical application of CAR T cell therapies.

## Introduction

Redirected chimeric antigen receptor (CAR) T cells (CART) have shown effective potency against hematologic tumors (1, 2). Second-generation CARs are hybrid receptors comprising a recognition domain, normally derived from a single-chain antibody fragment (scFv), fused to costimulatory, and cytotoxic signaling domains that enhance T cell function (3, 4). This restricts CAR T cells to recognize a single tumor antigen in a defined set of tumors, such as CD19 in B-cell malignancies. CD19-specific CAR T cell therapy for the treatment of CD19-positive B cell malignancies such as B-cell acute lymphoblastic leukemia (B-ALL), B-cell non-Hodgkin lymphoma (NHL), or chronic lymphocytic leukemia (CLL) has had remarkable success (5–8), resulting in their recent US Food and Drug Administration (FDA) approval. However, relapse of leukemia through CD19 loss variants in leukemia/lymphoma patients and immunosuppressive microenvironment or lack of tumor-associated antigens (TAAs) in solid tumors (9–11) represents major challenges for CAR T cell therapies. These inconveniences along with antigen-loss escape make it necessary to focus in other possible TAAs (9).

Natural killer group 2D (NKG2D) is an activating receptor expressed on different immune effector cells [natural killer (NK), CD8, and γδ T cells], although is in the NK cells where it has a main role in tumor surveillance. Ligands for NKG2D receptor, namely, MIC-A, MIC-B, and the UL-16 binding proteins, are expressed in 70% of human cancers including leukemia, osteosarcoma, or Ewing sarcoma (11–13), whereas their expression in healthy tissues is rare. We have produced a second-generation NKG2D CAR by fusing the full-length extracellular domain of human NKG2D to 4-1BB, which provides a costimulatory signal, and CD3ζ signaling domain. Thus, through the expression of this CAR, T cells acquire NK cell anti-tumor specificity while maintaining T-cell ability to expand and persist *in vivo*. The main advantages of this CAR are (1) the recognition of different ligands, widening clinical application, and potentially avoiding tumor immune escape by single antigen loss, and (2) it is a fully human CAR, causing less immunogenicity.

Most clinical trials use autologous T cells to express CARs; however, owing to low T-cell numbers, poor quality, or rapid disease progression, manufacturing of patient-derived CAR T cells is not always possible. To overcome these limitations, we propose here the use of allogeneic CAR T cells. Allogeneic cells expressing CARs have been infused into patients after a hematopoietic stem cell transplantation (HSCT) from the same healthy donor (14, 15). Nevertheless, the universal availability of large numbers of healthy donor T cells to express CARs and their infusion into patients without the requirement of a prior HSCT would be major challenges of CAR T cell immunotherapy. One potential risk of the use of allogeneic T cell-based therapies is the T-cell response against normal tissue: graft-vs.-host disease (GvHD). To avoid undesirable GvH reactions, T-cell products lacking an alloreactive T-cell receptor (TCR) are needed. Several methods have attempted to intensify graft-vs.-tumor (GvT) effects while minimizing GvH responses to lower toxicity and improve the outcome of treatment (14, 16, 17). One approach to enrich non-allogeneic T cells is by using antigen-experienced memory T cells for CAR transduction (16, 18). Predictably, the vast majority of T cells with a memory phenotype are likely to have encountered antigens other than the allogeneic type. Thus, selection for memory phenotype cells should enrich for a non-alloreactive repertoire. Indeed, memory T cells showed less potential to generate GvHD in murine models (19, 20), in part owing to non-alloreactive TCR enrichment along with the evidence that memory T cells are less likely to traffic to GvHD organs such as the gastrointestinal tract. Different extracellular markers can be used to differentiate naïve from memory T cells. Commonly, naïve T cells are CD45RA^+^CD45RO^−^CCR7^+^CD62L^+^, central memory T cells (T_CM_) are CD45RA^−^CD45RO^+^CCR7^+^CD62L^+^, effector memory T cells (T_EM_) are CD45RA^−^CD45RO^+^CCR7^−^CD62L^−^, and effector cells are CD45RA^+^CD45RO^−^CCR7^−^CD62L^−^ (21). Thus, one marker to roughly distinguish naïve from memory T cells is CD45RA (22). CD45RA is expressed on naïve T cells and a minor population of T memory stem cells (T_SCM_) (21), whereas CD45RO is expressed on memory T cells (22). CD45RA^+^ naïve T cells have high potential for alloreactivity against recipient-specific antigens upon adoptive transfer, causing clinical GvHD (23, 24). In contrast, CD45RA^−^CD45RO^+^ T cells exert a memory response to prior pathogens or vaccines and can mediate GvT effects without inducing GvHD (19, 25).

In the present study, we describe the manufacturing process to produce large-scale NKG2D CAR memory T cells from healthy donors for clinical use. In CAR T cell therapies, besides the designing of genetic constructs, the choice of effector cells to transduce, and the clinical trial design, the methods used to produce CAR T cells are key for clinical success. Thus, detailed description of each step along the manufacturing process and the full analysis of CAR T cell products composition at every step are essential. In fact, and according to the Good Manufacturing Practice (GMP) manufacturing standard, during the manufacturing procedure, in-process controls are carried out at different times. Optimization of manufacturing protocols to improve reproducibility, cost-effectiveness, and scalability will enable a broad application of CAR T cell therapies.

The NKG2D CAR memory T cells showed in this study were manufactured after 10–13 days of *ex vivo* processing, described in detail below, including activation with TransAct and IL-2, transduction with an NKG2D-CD8TM-4-1BB-CD3ζ lentiviral vector at multiplicity of infection (MOI) = 2, and expansion in CliniMACS Prodigy device. The NKG2D CAR memory T cells collected after this process fulfilled the release criteria with respect to safety, purity, and potency established in the protocols adhered to the guidelines of the current GMP (26–28). The manufacturing process developed in this study allows the automated GMP-compliant production of large doses of clinical-grade NKG2D CAR T cells in a short time and provides a robust and flexible base for further optimization of NKG2D CAR T cells manufacturing for their clinical application in different tumor types.

## Materials and Methods

### Starting Material

Non-mobilized apheresis was obtained from healthy donors at the Bone Marrow Transplant and Cell Therapy Unit (BMTCT) of Hospital Universitario La Paz (HULP) by using CliniMACS Plus device (Miltenyi Biotec). All donors gave their written informed consent in accordance with the Declaration of Helsinki protocol, and the study was performed according to the guidelines of the local ethics committee. All donors comply with the requirements regarding quality and safety for donation, obtaining, storage, distribution, and preservation of human cells and tissues under the Spanish specific regulation. CD45RA^+^ cells were depleted by immunomagnetic separation using CliniMACS CD45RA Reagent (701-46) and CliniMACS Plus system, both from Miltenyi Biotec, following manufacturer instructions. CD45RA^−^ cells were either processed immediately or stored at 2–8°C for subsequent processing no later than 24 h after depletion. The viability and purity of CD45RA^−^ fraction were analyzed by flow cytometry (FCM) before activation, transduction, and expansion.

### Construction and Production of Lentiviral Vector

The HL20i4r-MNDantiCD19bbz lentiviral vectors were derived from the clinical vector CL20i4r-EF1a-hgcOPT27 but expressed an NKG2D CAR. The anti-CD19-4-1BB-CD3ζ CAR designed by Imai et al. (29) was used as backbone to build the NKG2D CAR construct. It contained the extracellular domain of NKG2D (designed by Wai-Hang Leung and Wing Leung), the hinge region of CD8a, and the signaling domains of 4-1BB and CD3ζ. The cassette was driven by a prMND. The viral supernatant was produced according to GMP guidelines by transient transfection of HEK293T cells with the vector genome plasmid and lentiviral packaging helper plasmids pCAGG-HIVgpco, pCAGG-VSVG, and pCAG4-RTR2. Lentiviral plasmids were kindly provided by Dr. Byoung (St. Jude Children's Research Hospital). The virus supernatants were harvested and filtered through 0.22 μm filters. Virus supernatant was concentrated by ultracentrifugation and titrated on HeLa cells by serial dilution followed by a quantitative polymerase chain reaction (qPCR) to determine vector genome copy number.

### Manufacturing of Clinical-Grade NKG2D CAR T Cells

Activation, transduction, and expansion of CD45RA^−^ cells were performed on the CliniMACS Prodigy using Tubing set TS520 (170-076-600) and T-cell transduction (TCT) process. In detail, at day 0, cultivation was initiated with 10^8^ CD45RA^−^ cells in a total volume of 70 mL of TexMACs GMP medium (170-076-306) + 100 IU/mL of MACS GMP human recombinant IL-2 (170-076-147). MACS GMP TransAct CD3/CD28 Kit (170-076-156) was used for a 24-h activation at a final dilution of 1:17.5, as recommended by the manufacturer. At the following day, cells were transduced with NKG2D-4-1BB-CD3ζ lentiviral particles at MOI = 2. The vector was diluted in 10 mL of medium in a 150-mL transfer bag, which was attached to the CliniMACS Prodigy by sterile welding. The vector was automatically transferred in the culture chamber, and the vector bag was further rinsed with 20 mL of medium to bring the total culture volume to 100 mL. Residual TransAct was removed by an automated culture wash on day 4. Cells were then expanded for 10–13 days before being harvested. Sampling was performed at days +6 and +8 for in-process controls including cell counts, cytotoxicity, and FCM. At the end of the expansion, cells were automatically collected in 0.9% sodium chloride solution supplemented with 0.5% human serum albumin (Albutein 20%, Grifols) and transferred into a sterile bag. Release quality controls were performed at the end of the process.

### Analysis of Viability and Surface Immunophenotype by FCM

At day +6 and between days +8 and +10, as in-process controls, and at harvest, as release controls, NKG2D CAR memory T cell products were counted in a CELL-DYN Emerald hematology analyzer (Abbott) and analyzed for their viability, immunophenotype, NKG2D CAR expression, and activation status by FCM. The following anti-human fluorochrome-labeled monoclonal antibodies (mAb) were purchased from BioLegend: CD45RA-APC (Clone H100, 304111), CD3-PE/Cy7 (Clone HIT3a, 300316), CD4-APC/Cy7 (Clone OKT4, 317417), CD8 FITC (Clone KK1, 344703), NKG2D-PE (Clone 1D11, 320806), CD4 PerCP (Clone OKT4, 31743125), PD-1 APC (Clone EH12.2H7, 329907), Tim-3 APC Cy7 (Clone F38-2E2, 345025), CD25 APC (Clone BC96, 302610), and CD127 PE/Cy7 (Clone A019D5, 351320). Anti-human CD45RO-APC-Vio770 (Clone REA611, 130-114-083) was purchased from Miltenyi Biotec. Anti-human CCR7 PE (Clone 3D12, 552176) was purchased from BD Biosciences. The viability was tested by using DAPI or 7AAD as dead cell exclusion markers. Cells were analyzed using FACS CANTO II (BD Biosciences) and FlowJo v10.5.3 software (TreeStar). To ensure the expression of NKG2D CAR in the manufactured NKG2D CAR T cell products, we performed western blot with an antibody detecting CD3ζ. Total peripheral blood mononuclear cells (PBMC), activated and expanded NK cells (NKAE), untransduced CD45RA^−^, and NKG2D CAR T cells were pelleted and frozen at −80°C. Cell lysates were obtained by incubating cell pellets with RIPA (Millipore, 20188) supplemented with phosphatase inhibitor (PhosSTOP, 04906845001) and a cocktail of protease inhibitors (cOmplete Mini, 11836153001), both from Roche. Proteins were quantified using Bradford reagent (Bio-Rad Laboratories, 500-0205) and measuring absorbance at 595 nm in a Victor Plate Reader. Cell lysates were then mixed with the Laemmli sample buffer (Bio-Rad Laboratories, 161-0747), and equal amounts of protein (20 μg) were loaded on 4–15% Mini-PROTEAN TGX Gels (Bio-Rad Laboratories, 456-1086). Gels were transferred to polyvinylidene difluoride (PVDF) membranes. Blots were incubated with the mouse anti-human CD3ζ (BD Biosciences, 551033) or rabbit anti-human β-actin (Cell Signaling Technology, 4967S) primary antibodies at 4°C overnight. Horseradish peroxidase (HRP)-conjugated anti-mouse (Agilent, P0447) and anti-rabbit (Agilent, P0448) were used as secondary antibodies. The membranes were developed by enhanced chemiluminescence and exposed on Clarity Western ECL substrate (Bio-Rad Laboratories, 170-5060). The immunoblotting images were analyzed using the Image Lab software.

### Effector Function

The mechanism of action of CAR cells is complex, and there are no standardized methods to determine the degree of action. Although methods are available to determine potency, comparison of results is not easy owing to the absence of standardization (26). In our study, to test the cytotoxicity of manufactured NKG2D CAR T cells, conventional 4-h europium-TDA assays (PerkinElmer, AD0116) were performed as previously described (30) using a 20:1 effector to target ratio. The NKG2DL-expressing cell lines Jurkat and 531MII were used as targets. Cytotoxicity assays were performed on days +6 and +8 and at the end of the process. The 531MII primary osteosarcoma cell line was kindly provided by Dr. Patiño-García (Centro de Investigación Médica Aplicada (CIMA), Universidad de Navarra, Spain) and was cultured in minimum essential medium (MEM; GIBCO, 22571-020) supplemented with 10% heat-inactivated fetal bovine serum (FBS; GIBCO, 10270-098) and penicillin–streptomycin (P/S; GIBCO, 15140-122). The T-ALL Jurkat cell line was acquired from American Type Culture Collection (ATCC) and kept in culture in Roswell Park Memorial Institute (GIBCO, 61870-010) and 10% FBS and P/S. Both cell lines were routinely tested for mycoplasma.

### Analyses of Non-cellular Impurities

The detection of non-cellular impurities was carried out in accordance with the methodology recommendations of Chapter 2.6.21 and 2.6.7 of the European Pharmacopeia (Eu Ph) for mycoplasma and Chapter 2.6.14 for endotoxins. A DNA-binding dye-based qPCR system was employed for the detection of mycoplasma DNA in cell cultures. The assay was developed by the Genomics Unit in collaboration with the Monoclonal Antibodies Unit, both from CNIO, to detect 16s rRNA gene sequences from up to 70 *Mollicutes* species. Both specificity and sensitivity were extensively tested through benchmarking with established commercial systems (MTC-NI System, Millipore/GEN-PROBE Cat. No. 4573 and MycoAlert™ PLUS Mycoplasma Detection Kit, Lonza Cat. No. LT07-705). The Clinical Microbiology and Parasitology Service of HULP carried out the endotoxin test Endosafe-PTS (Charles River) to quantify endotoxin levels at day +8 and in final products.

### Microbiological Tests

At days +6 and between days +8 and +10 as in-process controls and at the end of manufacturing protocol, NKG2D CAR memory T cell products were tested for sterility according to Eu Ph 2.6.1. The microbiological tests were developed by the Clinical Microbiology and Parasitology Service of HULP by conventional microbiology techniques. In summary, sample tests were inoculated into separate culture media, and the growth of viable microorganisms was tested after several days. When a rapid result was required, Gram staining was used as a non-culture method, although it is a less sensitive technique than techniques based on culture (26).

### Genetic Tests, Genome Integrated Vector Copy Number, and Determination of Replication Competent Lentivirus in the Supernatant

Genetic tests and determination of vector copy number (VCN) and replication competent lentivirus (RCL) in the supernatant were carried out at days +6 and between days +8 and +10 as quality controls during process validation and at the end of the manufacturing process between days 10 and 13. To rule out chromosomal aberrations caused by lentiviral transduction, comparative genome hybridization (CGH) analysis was performed as previously described (30). Genome integrated lentiviral copy number and viral particles in supernatant were measured by qPCR according to Christodoulou et al. (31) using TaqMan Universal PCR Master Mix (Thermo Fisher; 4304437) and LightCycler 480 (Roche) after viral RNA extraction with RNeasy (Qiagen, 74104) and cDNA retrotranscription with Superscript II (Thermo Fisher, 18064014). The lack of oncogenic effects of the NKG2D CAR T cell products was verified using reverse transcriptase (RT)-PCR to detect c-*MYC* and telomerase (*TERT*) expression. Total RNA was isolated from the PBMCs using the RNeasy kit from Qiagen (PN 74104), followed by reverse transcription using SuperScript™ IV First-Strand Synthesis System from Thermo Fisher (PN 18091050). The resulting cDNA was amplified with the following specific TaqMan probes: Hs00972650_m1 (TERT), Hs00153408_m1 (MYC), and Hs02800695_m1 (HPRT1, housekeeping) from Life Technologies and the LightCycler 480 System from Roche. Finally, the data were analyzed by the comparative Ct methods as previously described (32). The genetic tests were performed at the Institute of Medical and Molecular Genetics of HULP (INGEMM).

### Effects of Cryopreservation on NKG2D CAR T Cells

As infusion of freshly manufactured CAR T cells is not always possible, we wanted to determine if cryopreservation could have a negative impact on viability, NKG2D CAR expression, and cytotoxicity of NKG2D CAR T cells. To this aim, spare CAR T cells were frozen at a concentration of 2.5–3 × 10^5^ cells/μL either by using HypoThermosol, M199 media supplemented with 10% human serum albumin and 5% dimethyl sulfoxide (DMSO), or in autologous plasma supplemented with 5% DMSO. One year after cryopreservation, NKG2D CAR T cells were thawed and evaluated for viability, NKG2D expression, and CD45RA^−^ purity by FCM and for cytotoxicity by europium-TDA as described above.

### Statistics Analyses

All statistical analyses in this study were performed using GraphPad Prism. Except indicated in another way, results are shown as median and interquartile range.

## Results

### Manufacturing Process: Activation, Transduction, and Expansion

**Figure 1:**
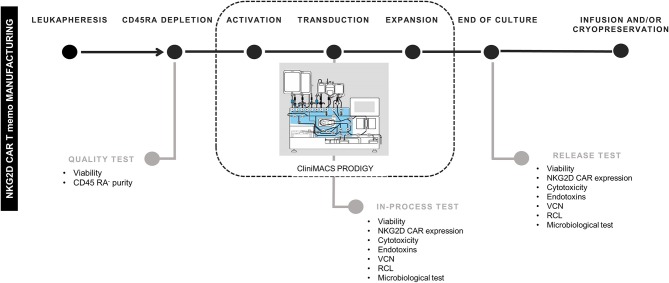
Schema of NKG2D CAR memory T-cell manufacturing process. NKG2D, Natural Killer Group 2D; CAR, chimeric antigen receptor. The schema of CliniMACS Prodigy is Copyrighted © 2015 by Miltenyi Biotec GmbH, used with permission.

CD45RA^−^ cells from four different donors were activated, transduced, and expanded in CliniMACS Prodigy in four different experiments. In-process tests were carried out at days +6 and +8. At the end of culture (between days +10 and +13), cells were harvested, and quality/release assays performed. A schema of the different steps for NKG2D CAR T cells manufacturing and the quality tests conducted along the process is shown in Figure 1.

### Purity of CD45RA^−^ Starting Cells

Non-mobilized apheresis from four different healthy donors were obtained and depleted for CD45RA^+^ cells at CliniMACS Plus. After depletion of CD45RA^+^ cells, median of purity of CD45RA^−^ population was 99.8 (range 99.7–99.9), and median of viability was 97.9 (range 97.7–99.9). Data of CD45RA^−^ purity and viability from each experiment are shown in Table 1.

**Table 1:** Purity and viability of CD45RA^−^ starting cells.

**Validation**	**% Viability**	**% of CD45RA^**−**^**
#1	98.1	99.8
#2	99.9	99.9
#3	97.7	99.9
#4	97.7	99.7

### Transduction Efficiency

**Figure 2:**
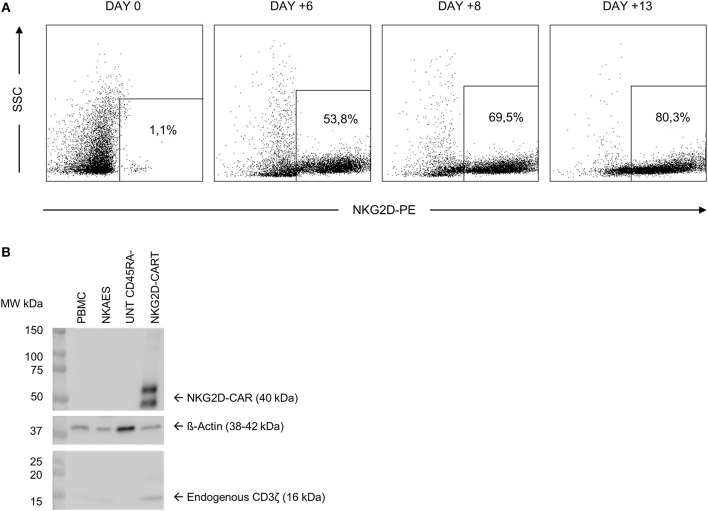
NKG2D CAR expression in CD45RA^−^ cells after transduction. **(A)** Representative FCM data showing an increase in NKG2D CAR expression along the manufacturing process. **(B)** Analysis of NKG2D CAR expression in transduced cells by western blot detecting CD3ζ. NKG2D, natural killer group 2D; CAR, chimeric antigen receptor; FCM, flow cytometry.

We transduced CD45RA^−^ cells 1 day after cell activation. A lentiviral construct encoding for NKG2D CAR was used at MOI = 2. As CD45RA^−^ cells only have basal levels of NKG2D receptor expression, we considered that the expression of NKG2D observed by FCM in NKG2D CAR T cell products corresponds to NKG2D CAR. Our target goal was to achieve ≥50% transduction of total cells. This goal was achieved for all four final products. Data from NKG2D CAR expression along the process are shown in Table 2. Representative dot plots of NKG2D staining at the different times are shown in Figure 2A. The anti-NKG2D antibody that we use for FCM does not discriminate between the NKG2D endogenous receptor and the NKG2D CAR. In order to analyze the expression of NKG2D CAR in the transduced cells, we performed a western blot using an anti-CD3ζ antibody to detect the CAR protein. NKG2D CAR protein is 40 kDa, whereas endogenous CD3ζ is 16 kDa. As shown in Figure 2B, bands corresponding to the NKG2D CAR were only observed in those cell lysates from transduced CD45RA^−^ cells, whereas they were absent in the different negative controls (activated and expanded NK cells, PBMC, and CD45RA^−^ untransduced cells). Additionally, PCR analysis using specific primers for endogenous NKG2D and NKG2D CAR genes further confirmed these results (Supplementary Figure 1).

**Table 2:** Data from transduction efficiency and viability.

	**% NKG2D CAR expression**			**Viability**		
**Validation**	**Day +6**	**Day +8**	**Final**	**Day +6**	**Day +8**	**Final**
#1	73	60.5	60.6	85	82.5	86.3
#2	41	43	55	73	77	65
#3	24	82	87.4	70	83	81.4
#4	62	75	91	80	84	82

*NKG2D, natural killer group 2D; CAR, chimeric antigen receptor*.

### Expansion

**Figure 3:**
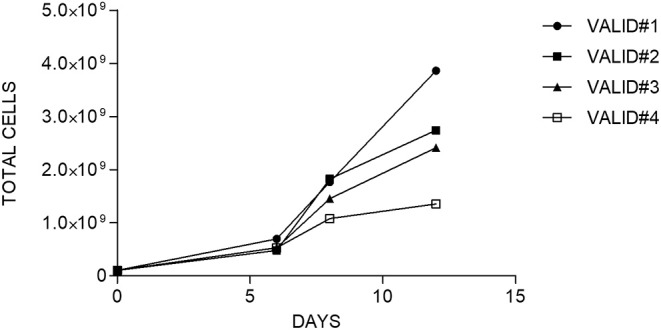
Fold expansion of cells along the time in each manufacturing validation.

After CD45RA^+^ depletion, 10^8^ of CD45RA^−^ cells were transferred into a sterile bag and connected to CliniMACS Prodigy for further processing. The number of cells recovered after CD45RA depletion exceeded this limit for all experiments. For the final products, the fold expansion ranged from 13.4 to 38.6; thus, in all cases, the total number of cells obtained was enough to perform a clinical treatment in a multiple-dose regimen. Data of cell expansion from each experiment are shown in Figure 3.

### Immunophenotype

**Figure 4:**
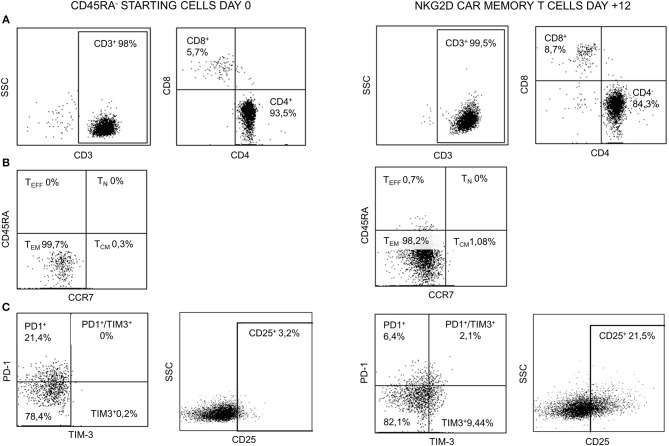
Representative FCM data of starting CD45RA^−^ cells (day 0) and NKG2D CAR memory T cell products at the end of manufacture process (day +12). **(A)** CD3, CD4, and CD8 contents. **(B)** Naïve/memory phenotype. **(C)** Expression of activation/exhaustion markers. FCM, flow cytometry; NKG2D, natural killer group 2D; CAR, chimeric antigen receptor.

Starting and final CAR T cell products were analyzed for viability and CD3, CD4, and CD8 contents. Naïve and memory populations were also identified by using CD45RA and CCR7 markers. The activation/exhaustion status of starting and final cells was analyzed by CD25, PD-1, and TIM-3 markers. The presence of CD4^+^CD25^+^CD127^low/neg^ (Tregs) was also analyzed in the starting and final products. Both starting CD45RA^−^ cells and final NKG2D CAR memory T cell products were CD3^+^ and showed an enrichment in CD4^+^ vs. CD8^+^ T cells. Before and after manufacturing process, T cells were negative for CD45RA and CCR7, indicating an effector memory (T_EM_) phenotype. Tim-3 and CD25 activation/exhaustion markers were upregulated in final NKG2D CAR memory T cell products compared to starting CD45RA^−^ cells; however, PD-1 expression was downregulated at the end of the process (Figure 4). Additionally, only a low proportion of T_regs_ (CD4^+^CD25^+^CD127^low/neg^) was found on starting CD45RA^−^ cells and final NKG2D CAR T cells compared with total PBMC (Supplementary Figure 2).

### Effector Function

Lysis ability of NKG2D CAR T cells was tested against the NKG2DL-expressing cell lines Jurkat (T-ALL) and 531MII (metastatic osteosarcoma) by performing conventional 4-h europium-TDA assays. Although donor variability was observed, all final NKG2D CAR T cell products analyzed could target Jurkat and 531MII cells with a percentage of cytotoxicity ≥20%, thus meeting the established requirements. For validation #1, owing to technical issues, cytotoxicity against Jurkat cells was only tested using cryopreserved NKG2D CAR T cells. Cytotoxicity of final NKG2D CAR T cells against Jurkat was higher (median 80%, range 28.2–100%) than against 531MII cells (median 42.3%, range 20–74.6%) for all analyzed products, although this difference was not statistically significant. Data of cytotoxicity levels from each experiment are shown in Table 3. Additionally, the cytotoxicity of manufactured NKG2D CAR T cells against Jurkat cells is shown in Supplementary Video 1.

**Table 3:** Cytotoxicity of NKG2D CAR memory T cells against Jurkat and 531MII target cells.

**Validation**	**% Cytotoxicity vs. Jurkat**	**% Cytotoxicity vs. 531MII**
#1	NR	74.6
#2	100	19.5
#3	79.8	42.3
#4	28.2	NR

*NKG2D, natural killer group 2D; CAR, chimeric antigen receptor; NR, non-reproducible experiment*.

### Safety and Purity Tests

To meet regulatory specifications (acceptable thresholds in parentheses), samples were taken at days +6 and between days +8 and +10 as in-process controls and at the end of process as release controls and were evaluated for VCN ( ≤ 5 copies/cell), free lentiviral particles in the supernatant (LVPS) ( ≤ 0.05%), oncogenic gene expression (no overexpression), and genetic stability (normal CGH). A Gram stain (no organisms seen) as a quick method and microbiological tests [0 colony-forming unit (CFU)] ensured no bacterial contamination. Other release controls performed included those relating to the purity of the final product: measurement of endotoxin levels, whose limits for administration depend on the product and the parenteral administration route, where the pyrogenic threshold dose of endotoxin per kilogram of body mass in a single hour in the case of transduced cells is 5 IU/kg/h. All final products analyzed fulfilled the specifications except for validation #3, which showed VCN of 12 instead of ≤5 copies/cell, and for validation 4#, which showed overexpression of myc. Complete data regarding genetic tests are shown in Table 4. All samples showed no microbiological contamination. Endotoxin levels were below 5 IU/kg/h, and the presence of mycoplasma was undetectable (Table 5).

**Table 4:** Results from genetic tests.

**Validation**	**VCN**	**LVPS**	**CGH**	***tert* expression**	***myc* expression**
#1	NA	Undetectable	Normal	No overexpression	No overexpression
#2	3.6	Undetectable	Normal	No overexpression	No overexpression
#3	12.3	Undetectable	Normal	No overexpression	No overexpression
#4	2.4	Undetectable	Normal	No overexpression	*myc* overexpression

*VCN, vector copy number; LVPS, free lentiviral particles in the supernatant; CGH, comparative genome hybridization*.

**Table 5:** Results from sterility tests.

**Validation**	**Gram staining**	**Mycoplasma**	**Endotoxins EU/mL**
#1	Negative	Negative	NA
#2	Negative	Negative	0.019
#3	Negative	Negative	0.0035
#4	Negative	Negative	0.01

### Stability of Cryopreserved NKG2D CAR T Cells

CliniMACS Prodigy allows the production of sufficient number of CAR T cells to be administered in multiple doses. Although the first dose could be administered right after harvesting, spare cells need to be cryopreserved for future infusions. To explore if cryopreservation could have a negative impact on manufactured CAR T cells, we tested three different freezing media and evaluated cell counts, viability CD45RA^−^ purity, NKG2D expression, and cytotoxicity of cryopreserved NKG2D CAR T cells 1 year after freezing. We observed that those NKG2D CAR T cells cryopreserved in autologous plasma +5% DMSO showed the highest viability and cytotoxicity indicating that, whenever possible, this should be the freezing media of preference, followed by M199 + 10% albumin and 5% DMSO (Table 6).

**Table 6:** Stability of manufactured NKG2D CAR memory T cells after cryopreservation.

**Freezing medium**	**% Viability**	**% NKG2D**	**% CD45RA^**−**^**	**% Cytotox** **vs. Jurkat**	**% Cytotox** **vs. 531MII**
M199 + ALB + DMSO V4	47.9	61.5	99.2	55.2	17.3
Hypothermosol v4	14.1	61.5	99.2	NA	NA
Auto plasma + DMSO V5	74.6	69.9	97.1	78.6	60.3

*NKG2D, natural killer group 2D; ALB, albumin; DMSO, dimethyl sulfoxide*.

## Discussion

In the last decades, CAR T cell-based immunotherapies have demonstrated to be an effective and safe approach for cancer treatment. The clinical success of two CART19 cell products (Kymriah™ and Yescarta™) for the treatment of B cell malignancies has led to their recent FDA and European Medicines Agency (EMA) approval, emphasizing the great potential of this technology. Targeting myeloid and non-B lymphoid cell hematological malignancies such as T-ALL, biphenotypic, and infant leukemia or solid tumors has been much harder, owing to the lack of specific antigens and the immunosuppressive tumor microenvironment (33–36). We present data on NKG2D CAR, which has the ability to recognize multiple ligands that are overexpressed in different tumor types including AML, T-ALL, and sarcomas (12, 13, 37–39). In fact, D. Sallman et al. have recently reported a case of remission in a patient suffering from relapsed/refractory AML after multiple infusions of autologous T cells redirected with a first-generation CAR recognizing NKG2DL (40). Aside from CAR specificity, choice of signaling domains and selection of effector cell subset to transduce, manufacturing process, and full characterization of final CAR T cell products are essential for clinical success. In the present study, we validate and provide detailed description of our manufacturing protocol and the characteristics of NKG2D CAR T cells. We show the feasibility of producing large numbers of allogeneic NKG2D CAR memory T cells using a 10–13 days' protocol, which includes activation with TransAct reagent, transduction with an NKG2D-4-1BB-CD3ζ lentiviral vector and expansion with rhIL-2 in CliniMACS Prodigy, an automated closed system compliant with GMP guidelines.

The main objectives of this study were (1) to show the feasibility and reproducibility of automated manufacturing of GMP-grade NKG2D CAR T cells in an academic institution and (2) to demonstrate that manufactured NKG2D CAR T cells meet the requirements established by the Spanish Regulatory Agency for clinical use. A total of four manufacturing processes were completed, and the CAR T cell products obtained were analyzed at three different time points: day +6 and between days +8 and +10, as in-process controls, and at the end of culture, between days 10 and 13, when quality tests were performed to analyze if NKG2D CAR memory T cell products met release criteria.

We used CD45RA^−^ cells from healthy donors to produce our NKG2D CAR T cells in order to develop a safe allogeneic therapy. The lack of alloreactivity of CD45RA^−^ cells has been explored before in preclinical studies from our group and others (18, 30). Furthermore, Maschan et al. have described the safety of low-dose infusions of CD45RA^−^ lymphocytes in mismatched-related HSCT (41). Recently, our group has reported the safety of high-dose infusions of donor-derived CD45RA^−^CD45RO^+^ T cells after haploidentical transplantation (42).

The manufacturing process starts with a non-mobilized apheresis from a healthy donor followed by depletion of CD45RA^+^ cells in CliniMACS Plus device. Although CD45RA^+^ cells can be currently depleted at CliniMACS Prodigy, at the moment when these experiments were carried out, the software to do so was unavailable. After depletion, CD45RA^−^ cells were tested for viability and purity. All CD45RA^−^ cell products showed viability >95%. Purity of CD45RA^−^ cells after depletion of CD45RA^+^ subset was at least 99.7%, indicating that carryover of naïve T cells was minimal and meets the established criteria for further processing to obtain NKG2D CAR T cells. Activation, transduction, and expansion were conducted in CliniMACS Prodigy. Median of fold expansion was 24.4 (range 13.5–38.6), and the mean of total cells obtained was 2.44 × 10^9^ (range 1.35 × 10^9^-3.86 × 10^9^). These expansion data are in line with other manufacturing protocols using CliniMACS Prodigy (43, 44). Additionally, according to the number of CAR T cells that have been infused in other clinical trials (45, 46), the number of NKG2D CAR T cells we achieved would have been enough to treat patients in a multiple-dose base. Over the manufacturing procedure, viability of the harvested cells has shown to be robust and above 80% except for validation 2, which showed a viability of 65%. During the process, a decrease in viability was observed on day +6 compared with that observed in starting cells, and this temporary drop on viability after transduction has been already reported by other groups (44, 47, 48). Some authors have reported that NKG2D CAR T cells may induce fratricide, hindering the expansion and the viability of cultured cells (49, 50). Additionally, a CD4/CD8 ratio bias and enhanced effector memory differentiation have been described when using PBMCs as starting cells to express NKG2D CAR. The fold NKG2D CAR T cell expansion observed in this study, along with the viability of the final cell products, suggests that no NKG2D CAR T cell-mediated fratricide is occurring during the manufacturing protocol. This observation could be related to the T-cell subset used as starting cells, as CD45RA^−^ compartment is already enriched in CD4^+^ T cells with an effector memory and central memory phenotypes. However, more experiments need to be performed to explore the susceptibility of different T-cell subsets to NKG2D CAR T cell-mediated fratricide to confirm this hypothesis. To further explore if fratricide could be taking place in our experiments, the expression of NKG2DL on NKG2D CAR T cells expanded at small scale was analyzed by FCM. No upregulation of NKG2DL was observed in these cells (data not shown). Nevertheless, we only analyzed the expression of NKG2DL at day +8 post-activation, and it has been described that activated T cells upregulate NKG2DL in a temporary manner, specially between days 2 and 5 upon activation (49, 50). Thus, with our data, we cannot totally rule out an upregulation of NKG2DL and, consequently, a fratricide phenomenon in other moments of the culture. A more detailed study of NKG2DL expression kinetics on NKG2D CAR T cells along the manufacturing procedure would shed light on this question.

Activated CD45RA^−^ cells were lentivirally transduced with MOI = 2 because small-scale preclinical data using the same vector achieved transduction efficiencies higher than 95% (30). This MOI of 2 is low compared with that of other works where MOIs of 5–10 are reported (43, 48). We used a fluorochrome-labeled anti-NKG2D mAb for CAR detection by FCM, as untransduced CD45RA^−^ cells only have basal expression of the NKG2D receptor, and then we can consider that the NKG2D expression observed in manufactured cells comes from the CAR (30). The expression of NKG2D CAR in transduced CD45RA^−^ cells was further confirmed by western blot and PCR analysis. As previously observed in small-scale experiments, the expression of NKG2D increased during the expansion of cells. In two out of four batches, NKG2D CAR expression was over 80%, whereas the other two achieved an expression of 55 and 60.6%. These expression values (55–60.6%) are comparable with those reported in other publications (43, 51) and were enough to efficiently eliminate Jurkat and primary osteosarcoma cells (531MII) at a 20:1 effector to target ratio. Owing to technical issues, some cytotoxicity assays were non-reproducible, and thus, potency of NKG2D CAR T cells could not be evaluated at some time points either during manufacturing procedure or at the end of culture. Nevertheless, those cytotoxicity assays that were reproducible also fulfilled the specification for potency, indicating manufactured NKG2D CAR T cells are cytotoxic against the target cells.

At the end of the activation–transduction–expansion protocol, different quality tests need to be performed to ensure safety and purity of manufactured CAR T cells before they are administered in patients. Sterility tests were negative, and no mycoplasma was detected. The concentration of bacterial endotoxins was within the limits set by Eu Ph for intravenous injectable products in all validations. Genetic stability of NKG2D CAR T was confirmed by normal CGH, indicating no chromosomal aberrations are caused by lentiviral transduction. Three out of four validations showed <5 genome integrated vector copies, fulfilling the specifications required. However, in validation #3, up to 12.3 genome integrated vector copies were detected. These data are striking, as a MOI of 2 was used in all experiments and does not match the hypothesis that one viral particle is able to infect one cell. Despite that higher-than-expected VCN was found in these cells, the percentage of NKG2D CAR positive cells in this validation was 87%, indicating transduction efficiency was not above the usual levels. Additionally, CGH and expression of *myc* and *tert* oncogenes were normal in this batch, suggesting that even though more than five copies were integrated, they caused no genetic alterations. To rule out a potential oncogenic effect of NKG2D CAR T cells, the expression of *myc* and *tert* oncogenes was analyzed. All validations showed no overexpression of these genes except for validation #4, which presented overexpression of *myc*, and consequently did not fulfill the specifications required. Although *myc* overexpression in NK cell products has been previously demonstrated to be safe and to induce no complications nor secondary neoplasia in patients (52), it would be important to be aware and increase monitoring of these cell products to ensure safety before being administered to patients. The specification of the percentage of RCL in the supernatants is established at a maximum of 0.05%. All NKG2D CAR T cell products remained under that limit, indicating that there is no potential risk of virus infection after infusion.

In summary, the data here reported demonstrate the feasibility and reproducibility of a manufacturing protocol to obtain clinical-grade large-scale NKG2D CAR CD45RA^−^ T cells in CliniMACS Prodigy system. NKG2D CAR T cells met the release criteria for expansion, NKG2D CAR expression, cytotoxicity, and sterility, although grade of expansion and product characteristics showed variability. Most importantly, the manufacturing process described here shows flexibility and admits further improvements for future NKG2D CAR T cell trials.

## Data Availability Statement

All datasets generated for this study are included in the manuscript/Supplementary Files.

## Ethics Statement

The studies involving human participants were reviewed and approved by The Ethics Committee from Hospital La Paz. Non-mobilized apheresis was obtained from healthy donors at the Bone Marrow Transplant and Cell Therapy Unit (BMTCT) of Hospital Universitario La Paz (HULP) by using CliniMACS Plus device (Miltenyi Biotec). All donors gave their written informed consent in accordance with the Helsinki protocol, and the study was performed according to the guidelines of the Ethics Committee from Hospital La Paz. The patients/participants provided their written informed consent to participate in this study.

## Author Contributions

LF, IM, and AP-M: conception and design. LF, AF, IM, DL, AM, and AR: development of methodology. LF, AE, MV, LC, AF, AL, MG, IM, RP, and AP-M: acquisition of data (acquired and managed patients, provided facilities, etc.). LF, AF, IM, AE, JM-L, and AP-M: analysis and interpretation of data (e.g., statistical analysis, biostatistics, computational analysis). LF, AF, IM, and AP-M: writing, review, and/or revision of the manuscript. LF, AF, IM, and AE: administrative, technical, or material support (i.e., reporting or organizing data, constructing databases). LF and AP-M: study supervision.

### Conflict of Interest

DL works for Miltenyi Biotec S.L. The remaining authors declare that the research was conducted in the absence of any commercial or financial relationships that could be construed as a potential conflict of interest.

## Supplementary Material

Supplementary Figure 1 PCR showing expression of endogenous NKG2D receptor and NKG2D CAR in transduced cells. NKG2D CAR expression is absent in CD45RA^−^ cells and PBMC negative controls.

Supplementary Figure 2 Representative FCM data of CD4^+^CD25^+^CD127^low/−^ (Treg) content in total PBMC from a healthy donor (first row), starting CD45RA^−^ cells from validation 2 (second row), and NKG2D CAR T cells from validation 4 at the end of manufacturing process (third row).

Supplementary Video 1 3.35 h Time-lapse showing how NKG2D CAR memory T cells (labeled in orange with CMTMR) recognize, bind, and eliminate Jurkat cells (labeled in green with CFSE).
