# Supplementary figures and images for: GMP-Compliant Manufacturing of NKG2D CAR Memory T Cells Using CliniMACS Prodigy
Source: Front Immunol. 2019 Oct 10;10:2361. doi: 10.3389/fimmu.2019.02361 (PMC6795760; doi:10.3389/fimmu.2019.02361)

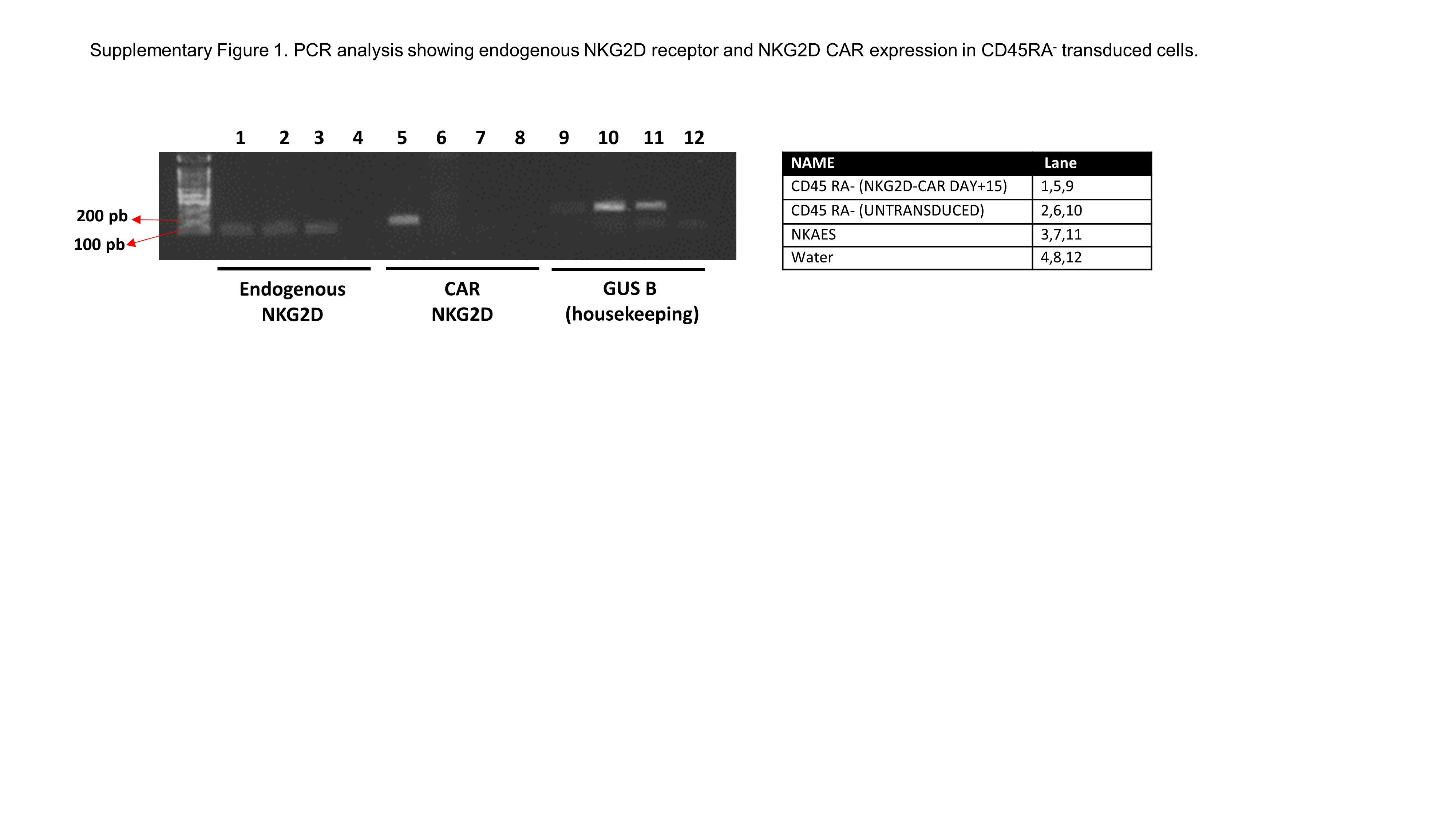

Supplement: Supplementary Figure 1 — PCR showing expression of endogenous NKG2D receptor and NKG2D CAR in transduced cells. NKG2D CAR expression is absent in CD45RA− cells and PBMC negative controls. [file Image_1.TIF]

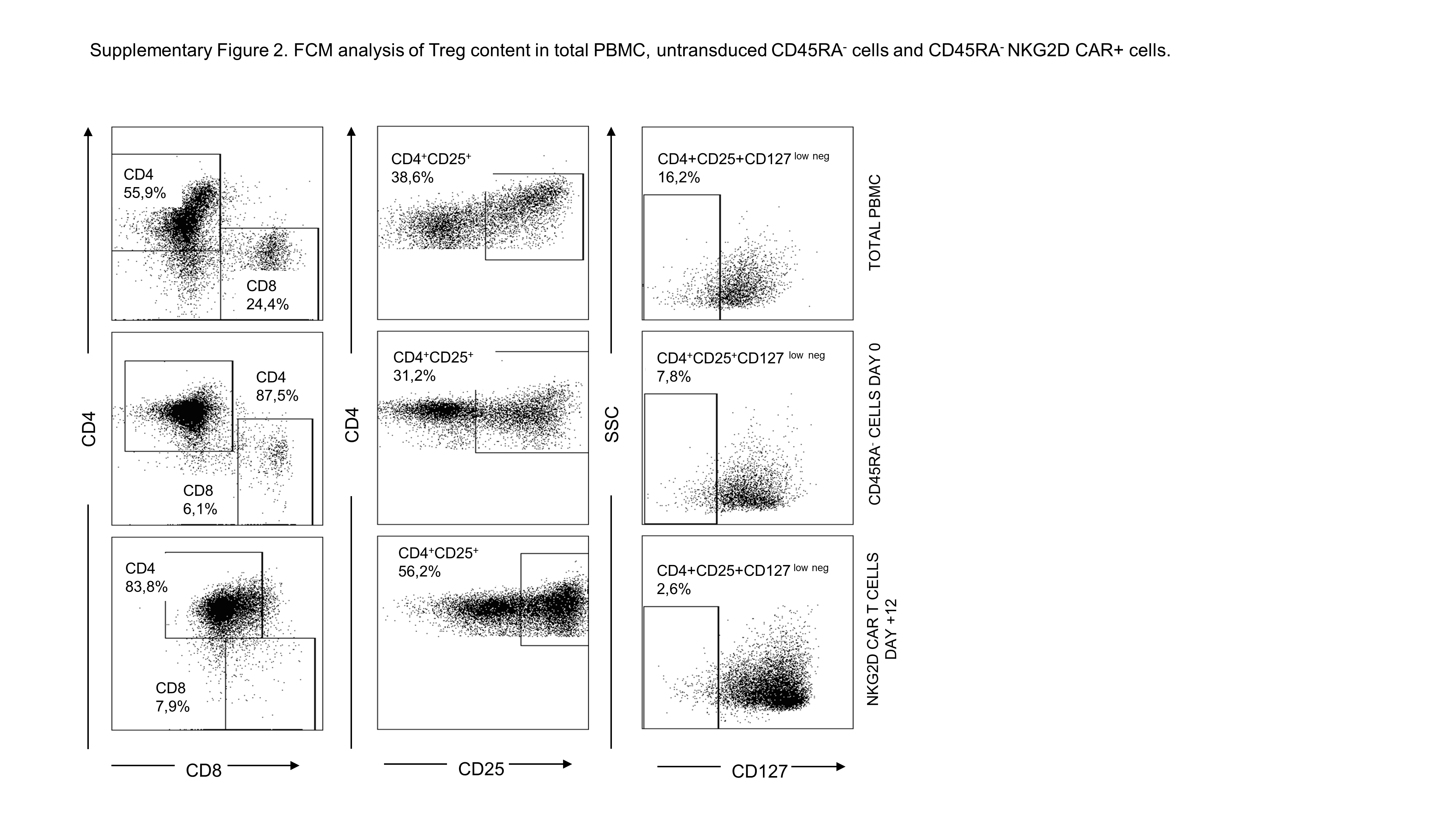

Supplement: Supplementary Figure 2 — Representative FCM data of CD4+CD25+CD127low/− (Treg) content in total PBMC from a healthy donor (first row), starting CD45RA− cells from validation 2 (second row), and NKG2D CAR T cells from validation 4 at the end of manufacturing process (third row). [file Image_2.TIF]
